# Dendritic cells during Staphylococcus aureus infection: subsets and roles

**DOI:** 10.1186/s12967-014-0358-z

**Published:** 2014-12-18

**Authors:** Xuejie Wu, Feng Xu

**Affiliations:** Department of Infectious Diseases, Second Affiliated Hospital, Zhejiang University School of Medicine, Hangzhou, 310009 China

**Keywords:** Dendritic cells, Pattern recognition receptors, Toll-like receptors, *Staphylococcus aureus*

## Abstract

Dendritic cells (DCs) are professional antigen-presenting cells (APCs) that play a crucial role in both innate and adaptive immune responses. DCs orient the immune responses by modulating the balance between protective immunity to pathogens and tolerance to self-antigens. *Staphylococcus aureus (S. aureus)* is a common member of human skin microbiota and can cause severe infections with significant morbidity and mortality. Protective immunity to pathogens by DCs is required for clearance of *S. aureus.* DCs sense the presence of the staphylococcal components using pattern recognition receptors (PRRs) and then orchestrate immune systems to resolve infections. This review summarizes the possible roles of DCs, in particular their Toll-like receptors (TLRs) involved in *S. aureus* infection and strategies by which the pathogen affects activation and function of DCs.

## Introduction

Dendritic cells (DCs) were first identified by Steinman and colleagues in the early 1970s. The nomenclature was originally based on the cell’s stellate shape and membranous processes [1]. DCs are now recognized as one of the most potent and efficient professional APCs within the immune system [2]. They have the unique capacity to prime naïve T cell-mediated protective immune responses as well as maintain tolerance to self-antigens by controlling antigen presentation to T cells [3]. Upon antigen uptake and exposure to appropriate proinflammatory signals or cellular stress, DCs generally migrate from the tissue to secondary lymphoid organs and differentiate into mature DCs which are characterized by upregulation of major histocompatibility complex (MHC) class I and II, adhesins and costimulatory molecules. Here, they process and present captured antigens in the presence of the correct costimulatory molecules to initiate antigen-specific T and B cell immune responses [4,5]. DCs also participate in inducing and maintaining peripheral immune tolerance in the steady state, possibly through the induction of T-cell anergy or T regulatory cells (Tregs) via self-antigen presentation [6,7].

In the past decades, huge efforts have been made to explore the myriad immune functions of DCs after the pioneering work initiated by Steinman and colleagues. It is now well known that the DCs represent a heterogeneous cell population with distinctive functions and subsets [8]. Generally, human and mouse DCs can be grouped into at least two subsets, referred to as conventional DCs (cDCs) and plasmacytoid DCs (pDCs) based on differing morphological, phenotypic and functional specificity. cDCs can be further subdivided into two main subsets according to their phenotype, functional specialization, specific gene expression program and transcription factors [9-11]. These subdivisions correlate to the large and differing roles that DCs have on every aspect of the immune system, and will be described in detail below.

*S. aureus* is a common member of human skin microbiota and is well habituated to the human host [12,13]. This pathogen can cause infections of nearly all tissues and organs, and staphylococcal disease can vary in severity ranging from skin and soft tissue infections to necrotizing fasciitis, pneumonia, endocarditis, sepsis, and toxic shock syndrome. The high pathogenecity of *S. aureus* depends on its numerous tactics to evade immune system [14-17]. At present, infection of methicillin-resistant *S. aureus* (MRSA) which is resistant to most of the current available potent antibiotics poses a serious public health threat [18,19]. MRSA was previously confined to hospital settings where antibiotics have been used in abundance and the chance to make contact with this bacterium is very high [20]. However, community acquired MRSA (CA-MRSA) has emerged soon and gained much attention by clinicians and investigators [16,21].

DCs sense the presence of pathogens using pattern recognition receptors (PRRs), which recognize pathogen associated molecular patterns (PAMPs) expressed by various microorganisms. TLRs are the most well described PRRs [22]. To date, 13 different TLRs have been identified, 10 human TLRs (TLR1-10) and 12 mouse TLRs (TLR1-9, TLR11, TLR12, TLR13). TLR10 and TLR11-13 are specific to humans or mice respectively [23,24]. TLR 1, 2, 4-6 and 11 are expressed at the cell surface while TLR 3 and 7-9 are located in the endosomal compartments [25]. It has recently been shown that TLR-driven early glycolytic reprogramming is essential for the anabolic demands of DC activation and function [26]. TLR expression varies according to DC subset. In humans, cDCs express TLR 1-8 and 10, and can be activated by various bacterial products [27,28]. Conversely, human and mouse pDCs express a restricted set of TLRs including TLR7 and TLR9 which sense viral RNA and DNA respectively [29,30]. Several TLRs have been implicated in the recognition of staphylococcal structures. For example, TLR2 is involved in the recognition of staphylococcal peptidoglycan, lipoteichoic acid (LTA) and staphylococcal enterotoxin B (SEB), while TLR4 can be activated by leukocidin (Luk), an exotoxin of *S. aureus* [31-34]. The differing TLRs of DC subsets and corresponding ligands are summarized in Table 1.

**Table 1:** TLRs of DC subsets and corresponding ligands

**DC subsets**	**TLR**	**ligands**
cDCs	1/2	Triacyl lipoprotein
	2	Peptidoglycan, Lipoteichoic acid
	3	dsRNA
	4	Lipopolysaccharide
	5	Flagellin
	6/2	Diacyl lipoprotein
	7	ssRNA
	8	ssRNA
	10	No ligand known
pDCs	7	ssRNA
	9	unmethylated CpG motifs of DNA
	10	No ligand known

Abbreviations: dsRNA, double-stranded RNA; ssRNA, single-stranded RNA.

This review will focus on the possible roles of DCs and their well-equipped armamentarium, particularly TLRs in *S. aureus* infection, as well as the strategies utilized by *S. aureus* to evade DCs. Importantly, the findings summarized here may have implications for developing novel therapies aimed at improving DC function during *S. aureus* infection.

## Dendritic cell subsets and TLRs

### cDCs and their TLRs

cDCs were initially thought to be of myeloid origin, based on their expression of the myeloid markers, such as CD13 and CD33 [35,36]. However, several studies have shown that cDCs may also originate from lymphoid precursor cells expressing Flt3 under the treatment of Flt3 ligand (Flt3L), a growth factor for hemopoietic progenitors [37-39]. Murine cDCs include both lymphoid organ-resident DCs and migratory DCs [40]. Considering the complexity of the cDC origin, most studies simply categorize murine cDCs as CD11b^+^ or CD8α^+^/CD103^+^ cDCs. Here, CD8α^+^ DCs are demonstrated to be more efficient in the cross-presentation of exogenous antigens on MHC class I molecules [2,41]. In humans, cDCs can be further identified as BDCA1^+^DCs and BDCA3^+^DCs according to the characteristic surface expression of BDCA1 (CD1c) and BDCA3 (CD141) [42-44]. Human cDCs nearly express all TLRs except TLR9 [28,29,45,46]. Although cDCs do express TLR10, its ligands and functionality are still unknown [47]. Extracellular TLRs of cDCs such as TLR1, 2, 4-6 recognize cell wall components of bacteria and fungi. Lipopolysaccharide (LPS), a structurally diverse molecule, is a well-known ligand for TLR4 and the mechanisms involved in versatility of ligand recognition which is crucial for combating diverse microbial infection is unraveled by TLR4-MD-2-LPS structure [48]. TLR5 activation by flagellin is important for priming of the adaptive immune response in the intestine [49]. Except for direct recognition of the Gram positive bacteria-derived peptidoglycan [50], triacyl lipoprotein and diacyl lipoprotein can also be recognized by TLR2 through the formation of heterodimers with TLR1 or TLR6 respectively [22]. This exemplifies the complexity of TLRs and PAMPs recognition. After being activated by TLR ligands or agonists, cDCs secrete several cytokines important in immune responses, such as IL-6, IL-8, IL-10, IL-12 and TNF-α [22,51]. Besides, cDCs also recognize nucleotide-containing structures via intracellular TLRs. For example, TLR3 recognizes virus-derived double-stranded RNA (dsRNA) while TLR7 and TLR8 recognize single-stranded RNA (ssRNA) which comes from bacteria, virus or self [22,52,53].

### pDC**s and their TLRs**

Cell surface markers of human pDCs are characterized as CD4^+^CD45RA^+^HLA-DR^+^ CD123^high^CD11c-lineage-cells. pDCs do not express most of the myeloid antigens, such as CD11b, CD13, CD14, or CD33 and were suggested to be of lymphoid origin. Human pDCs express two additional markers, BDCA2 (CD303) and BDCA4 (CD304) [39,54]. It is now known that both human and mouse pDCs may also stem from myeloid precursors expressing Flt3 when stimulated with Flt3L [37,38]. Compared to cDCs, both human and mouse pDCs preferentially express high levels of TLR7 and TLR9 [29,45]. Although minimal expression of TLR1 on the pDCs has been reported by several studies, its biological function remains unclear [29,47,55]. More importantly, pDCs neither express TLR2, a heterodimeric partner for TLR1, nor respond to TLR1/TLR2 ligands [29,45,55,56]. pDCs also express TLR10 which has been confirmed at both the mRNA and protein level [47,55,57]. However, the biological function and ligand of TLR10 have not been determined. pDCs preferentially secrete type I IFNs, especially IFN-α when TLR7 and TLR9 are triggered by ssRNA and unmethylated CpG motifs of DNA, respectively [28,29,45,55,58].

**Figure 1:**
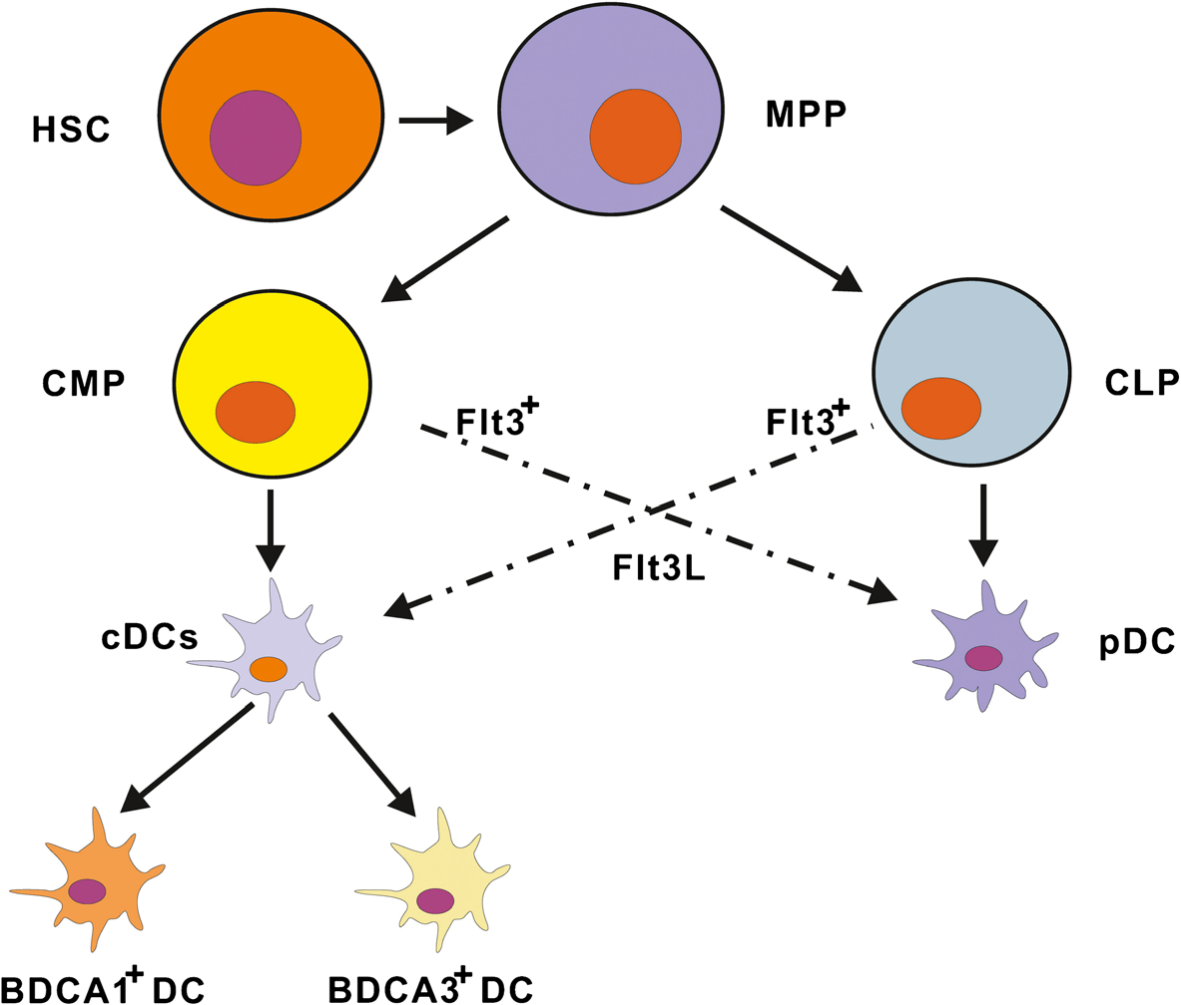
**A developmental scheme for human DC lineages.** Both cDCs and pDCs can originate from CMP and CLP lineages. Human cDCs are categorized as two main subsets including BDCA1^+^DCs and BDCA3^+^DCs. Abbreviations: HSC, hemopoietic stem cell; MPP, multipotent progenitors; CMP, common myeloid precursors; CLP, common lymphoid precursors. cDCs, conventional DCs; pDCs, plasmacytoid DCs.

Although DCs are rare subsets of cells among all blood mononuclear cells, they serve as a bridge linking innate and adaptive immune responses. Current data suggest that both cDCs and pDCs can be either myeloid or lymphoid origin. A developmental scheme for human DC lineages is shown in Figure 1.

### Virulence factors of *S. aureus*

The whole genome of *S. aureus* encodes numerous toxins, mainly including pore-forming toxins, exfoliative toxins and superantigens [59]. The most common pore-forming toxins are hemolysin-α (Hla, α-toxin), leukotoxins and phenol-soluble modulins (PSMs) [60,61]. Hla is released by most *S. aureus* clinical isolates and spontaneously assembles into a heptameric pore in target cell membranes. It preferentially targets human lymphocytes and monocytes but not neutrophils [62,63]. Leukotoxins belong to a bicomponent family of cytotoxins consisting of one class S and one class F protein [64]. To date, five class F subunits (HlgB, LukF-PV, LukD, LukF’-PV, and LukG) and six class S subunits (HlgA, HlgC, LukS-PV, LukE, LukM, and LukH) have been reported [60]. Leukotoxins can lyse monocytes, macrophages, and neutrophils via forming hetero-oligomeric pore complexes in target cell membranes [65,66]. PSMs, including the PSMα and PSMβ subfamilies are small, α-helical amphipathic peptides which have multiple roles in staphylococcal virulence [67,68].

Exfoliative toxins (ETs) A, B and D produced by *S. aureus* can efficiently cleave a single peptide bond in the extracellular region of human and mouse desmoglein 1 (Dsg1) [69]. The expression of ETs has been confirmed to be regulated by accessory gene regulator (agr) [70]. ETs are glutamate-specific serine proteases with high species specificity and responsible for staphylococcal scalded skin syndrome (SSSS), which predominantly affects infants and is characterized by the loss of superficial skin layers, dehydration, and secondary infections [71].

The staphylococcal superantigens (SAgs) are a family of potent immunostimulatory exotoxins. At present, 23 distinct staphylococcal SAgs have been characterized: the staphylococcal enterotoxins (SEs) A, B (multiple variant forms exist), C (multiple minor variant forms exist), D, E, and G; the staphylococcal enterotoxin-like toxins H, I, J, K, L, M, N, O, P, Q, R, S, T, U, V, and X and toxic shock syndrome toxin-1 (TSST-1) [60,72,73]. Some of them can directly cross-link MHC class II molecules on APCs with T cell receptors to induce potent T-cell activation and cytokine release [74,75].

### Roles of different subsets of DCs in *S. aureus* infection

DCs are more potent in presenting small amounts of microbial superantigens such as SEA [76], B, E and TSST-1, compared with monocytes or B cells. They also stimulate T cell proliferation far more efficiently than monocytes or B cells in the presence of SEA, SEB, SEE and TSST-1 [77], all of which bind well to MHC class II molecules [78-80]. After stimulation with the *S. aureus* Cowan strain I, both mouse and human DCs expressed IL-12 p40 and p35 mRNA. More importantly, they also produced IL-12p70 protein, a crucial factor for induction of Th1 immune response [81]. The *S. aureus* Cowan I strain, when cultured with epidermal DCs from the skin, also known as Langerhans cells (LCs), induced IL-6 (inflammatory cytokine), IL-12p40 (Th1 cytokine) production and inhibited TARC (Th2 cytokine) production by LCs [82]. When stimulated with SEB alone, DCs secreted abundant TNF-α and slight IL-12. However, IL-1β and IL-10 were undetectable in the culture medium. Concurrent with this process, DCs also expressed significantly higher HLA-DR and costimulatory molecules [83]. Although DCs do not kill *S. aureus* directly, depletion of DCs in CD11c- DTR transgenic mice resulted in increased bacterial loads in kidneys and lungs, higher mortality, more severe inflammatory injury and abolishment of IL-12 production. Nevertheless, adoptive transfer of either immature or LPS-matured bone marrow-derived DCs into normal BALB/c mice improved the capacity of these animals to eliminate *S. aureus* bacteria in the lungs. In addition, the impaired immunity of DC-depleted mice to *S. aureus* can be recovered by using exogenous recombinant mouse IL-12 [84]. These studies suggest that DCs preferentially induce Th1 immune response when encountered with *S. aureus* or SEs, probably through producing of IL-12, and then orchestrate innate and adaptive immune responses to fight against *S. aureus* infection.

In addition to SEs, DCs also react to other components derived from *S. aureus*. Leukocidin (Luk), an exotoxin of *S. aureus* is also capable of initiating the Th1-oriented immune response. Inden et al. demonstrated that LukF was the main mediator to induce IL-12p40 and TNF-α production in DCs in a dose-dependent fashion. Using a TLR4-deficient mutant mouse model, IL-12p40 production of DCs triggered by LukF was found to be controlled by TLR4 signaling [34]. In contrast to monocytes and monocyte-derived macrophages which preferentially prime modulatory IL-10 and weak IL-17 responses, monocyte-derived DCs were found to produce mainly IL-12 and IL-23, and initiate robust Th1/Th17 responses after staphylococcal peptidoglycan stimulation via TLR2 signaling [85,86]. Additionally, DCs can also be activated by lipoproteins of viable *S. aureus* through TLR2-MyD88 signaling and secret more IFN-γ and IL-17 in CD4^+^ T cells to improve bacterial clearing and disease outcome [87].

However, DCs may also have a role in the worsening of atopic dermatitis (AD) inflammation caused by secondary *S. aureus* infection. Wash fluid from AD lesions with secondary *S. aureus* infection induced DCs to secrete proinflammatory cytokines including IL-1β, IL-6, IL-10 and TNF-α in a MyD88-dependent manner. Production of these cytokines strongly correlated with wash fluid-contained lipoteichoic acid (LTA) which is a known microbial ligand derived from *S. aureus* for TLR2 activation [88,89]. Further study showed that staphylococcal LTA and muramyl dipeptide, the minimal structural unit of peptidoglycan with immunostimulating activity, synergistically induced maturation of human DCs and secretion of TNF-α and IL-12 p40 [90]. In order to unravel the roles of different DC subsets in *S. aureus* infection in detail, Jin and colleagues compared the differences of CD16^+^ and BDCA3^+^ DCs, BDCA1^+^ DCs treated with *S. aureus*. In contrast to CD16^+^ and BDCA3^+^ DCs, BDCA1^+^ DCs expressed high levels of TLR2 and scavenger receptor A (SR-A) which are required for recognition of *S. aureus* and subsequent activation of BDCA1^+^ DCs. Consistent with previous studies, BDCA1^+^DCs engulfed *S. aureus* efficiently and then dramatically upregulated expression of surface markers, such as costimulatory molecules, MHC class I and II molecules. Moreover, BDCA1^+^DCs also secreted abundant proinflammatory cytokines and promoted IFN-γ production in CD4 and CD8 T cells [91]. These data indicate that DCs have a complicated role in *S. aureus* infection. It is appropriate to speculate that distinct subsets of DCs may fulfill different immune responses against *S. aureus* infection. These differences may explain the diverse outcomes of humans infected with *S. aureus*.

**Figure 2:**
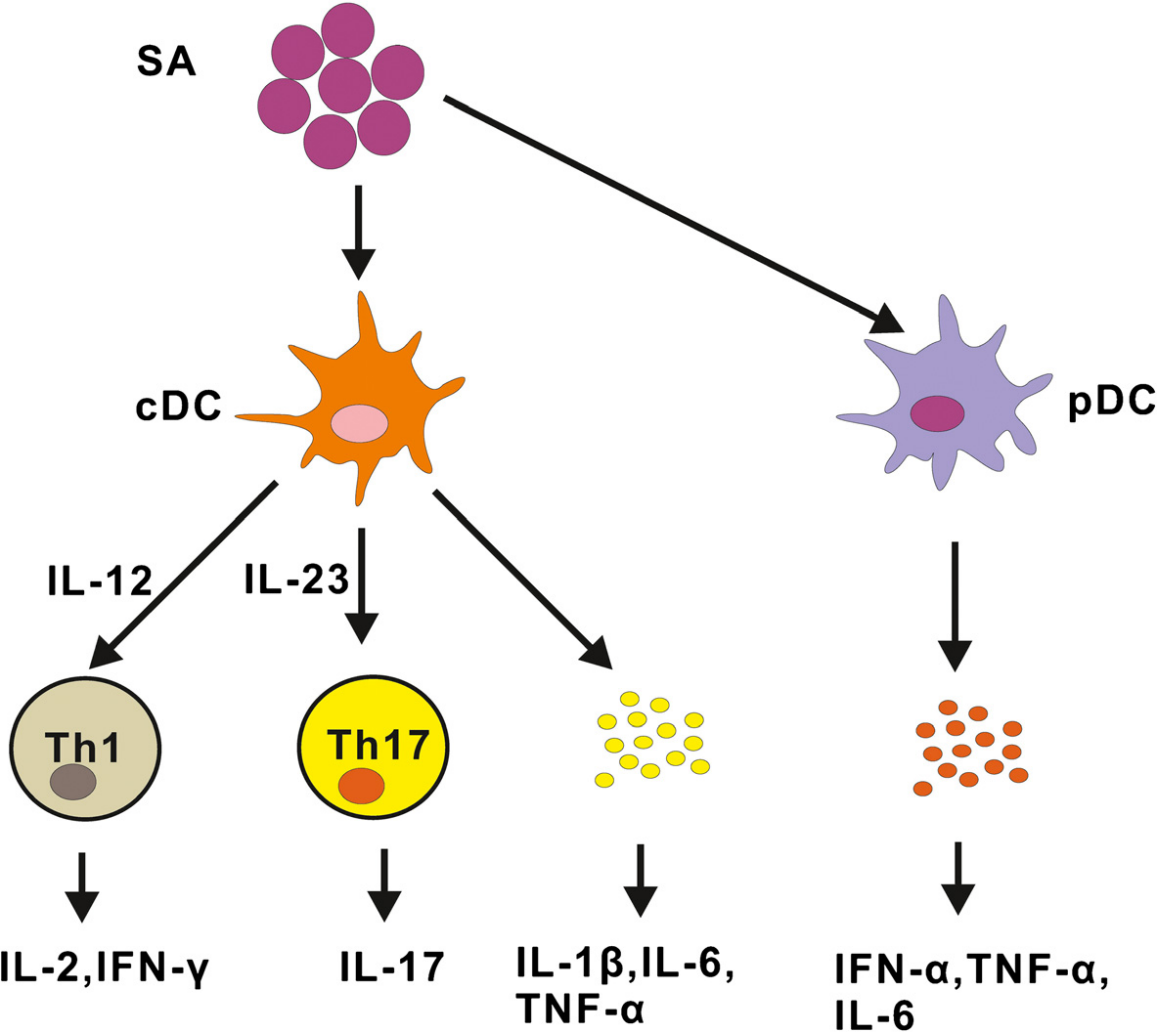
**Possible roles of DCs in** ***S. aureus*** **infection.** *S. aureus* triggered DCs (cDCs and pDCs) secret a variety of cytokines which are vital in inducing protective immune responses against *S.aureus* as well as promoting inflammation. Th1, Th17 cells and cytokines produced by these effector cells are involved in this process. Abbreviations: SA, *S. aureus*.

pDCs, in addition to their well established involvement in the viral immune response [39], also take part in host response to extracellular bacteria, including *S. aureus*. Parcina et al. showed that IFN-α production of pDCs triggered by *S. aureus* was independent of TLR2 and specific for coagulase-positive staphylococci. The specificity of the pDCs response to *S. aureus* was mediated by Ag-specific IgG and CD32. Blockade of TLR7/9 using inhibitory DNA oligonucleotides and chloroquine abrogated *S. aureus*-induced pDC activation, indicating the involvement of TLR7/9 by bacterial nucleic acids in this process [92]. It is reported that pDCs are located in tonsillar crypts and oro-nasopharyngeal epithelium, where they may encounter extracellular bacteria including colonized *S. aureus*. These pDCs do produce abundant IFN-α, TNF-α, IL-6 and show upregulated CD86 expression when activated by *S. aureus* [93]. However, some studies reported that pDCs alone are unresponsive to extracellular bacteria including *S. aureus* [94,95]. Further studies are needed to clarify the function of pDCs during *S. aureus* infection. Possible roles of DCs in *S. aureus* infection are illustrated in Figure 2.

### Strategies of *S. aureus* to affect DC function

Skin sections treated with SEA or exfoliative toxin lead to significant depletion of LCs. Cholera and pertussis toxin-sensitive GTP-binding proteins were found to be involved in the LC depletion in response to SEA [96]. Muraille et al. demonstrated that APCs, a mixture of B cells, macrophages and DCs, obtained from mice injected 2 days earlier with SEB failed to stimulate an alloreactive *in vitro* response. In spite of an increase in spleen cellularity secondary to SEB injection, the absolute and relative number of DCs was significantly decreased upon SEB-treatment. However, no significant reduction was observed in splenic B cells and cells expressing a typical macrophage phenotype [97]. Further study showed that DCs activated by SEB secreted high levels of IL-2 but no IL-12p70 and drove polarization of naïve T cells into the Th2 subset. Using the TLR2 stably transfected human embryonic kidney (HEK) 293 cells and anti-TLR2 antibodies, the signaling involved in this process was demonstrated to be triggered by the SEB-TLR2 interaction [32]. SEB also promoted the expression of T cell immunoglobulin mucin domain (TIM) 4 in human DCs. TIM4 expressed-DCs preferentially induced Th2 responses through the interaction of TIM4 and TIM1 [98].

Phenol-soluble modulin (PSM) peptide toxins are strongly expressed in CA-MRSA and exert their function on DCs through interacting with the formyl peptide receptor (FPR)2. During infection, PSMs, a virulence factor of CA-MRSA, attracted DCs through interaction with FPR2. PSMs treated-DCs showed impaired clathrin-mediated endocytosis and reduced secretion of the proinflammatory cytokines such as TNF, IL-12 and IL-6 while increased IL-10 secretion. The IL-10 producing-DCs inhibited T-cell activation and T-cell priming towards Th1 cells. Furthermore, they also induced IL-10 producing Tregs *in vitro* [99]. In turn, Tregs can also provide DCs with immune regulatory activity in mice unresponsive to SEB through IFN-γ-independent CD152-mediated activation of tryptophan catabolism [100]. These results suggest that *S. aureus* can also direct DCs to induce Th2 and Treg cell responses but inhibit Th1 responses in certain conditions. However, the accurate mechanisms of DCs to initiate different immune responses have not been determined currently.

Apart from its effect on DC function, *S. aureus* can also directly kill DCs to avoid immune clearance. Dumont et al. demonstrated that a novel styphylococcal cytotoxin denoted leukocidin A/B (LukAB) was responsible for cytotoxicity towards monoctye-derived DCs. Although LukA and LukB alone have little effect on cell viability, a combination of the purified recombinant LukA and LukB potently kills DCs [65]. Additionally, CCR5, a well-known HIV coreceptor has also been confirmed as a receptor for the *S. aureus* leukotoxin ED. Through its interaction with CCR5, leukotoxin ED selectively kills CCR5^+^ T lymphocytes and myeloid cells including monocyte-derived DCs in a CCR5-dependent manner [101].

**Figure 3:**
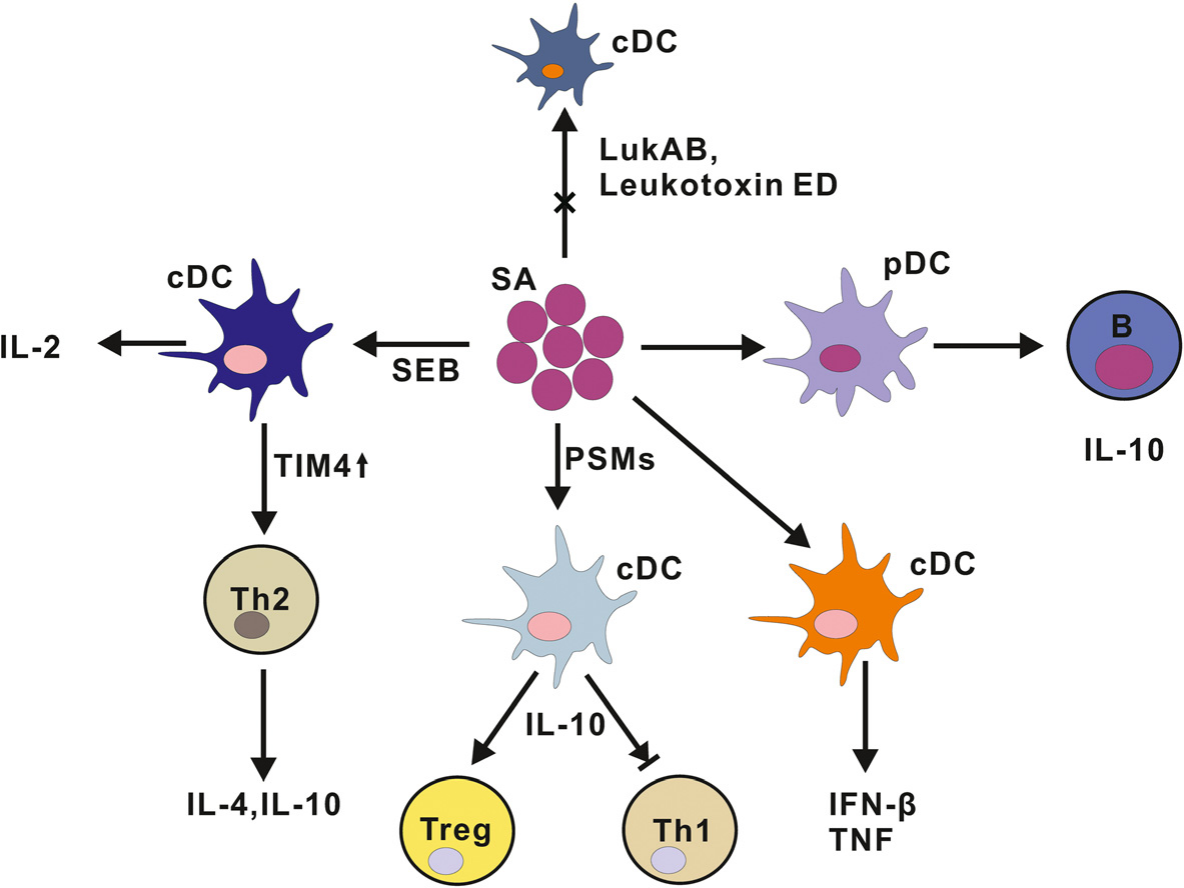
**Possible strategies of** ***S. aureus*** **to affect DCs function.** Several components of *S. aureus* may affect DCs function. Possible tactics used by *S. aureus* include killing DC directly, inhibiting Th1 responses, inducing Th2 and Treg responses as well as IL-10-producing B cells. Abbreviations: SEB, *S. aureus* enterotoxin B; TIM4, T cell immunoglobulin mucin domain (TIM) 4; LukAB, leukocidin A/B; PSMs, Phenol-soluble modulin; spA-SA, surface protein A-bearing *S. aureus*.

*In vitro* studies showed that bone marrow derived DCs of TLR9^-/-^ mice produced significantly decreased IFN-β levels after incubation with *S. aureus* USA300 for 20 hours, compared to wildtype mice. However, TLR9^-/-^ mice exerted enhanced clearance of *S. aureus* from the airways and lung tissue. Further analysis indicated that TNF may be related to the control of *S. aureus* infection because its level in BALF was reduced significantly in infected TLR9^-/-^ while the other proinflammatory cytokines including IL-17, CXCL-10, KC, IL-6, and IFN-γ were unchanged, compared to controls [102]. This is consistent with the detrimental effect of TNF signaling on *S. aureus* pulmonary infection [103]. Parcina and colleagues showed that human pDCs activated by surface protein A-bearing *S. aureus* were involved in TLR9 signaling and mediated B cell proliferation and Ig production. Cooperation of pDCs and B cells enhanced B cell-derived IL-10 production of which was partially dependent on TLR2-active lipoproteins, a hallmark of the Staphylococcus species [104]. These results indicate that *S. aureus* may exploit pDCs and their TLRs to establish B cell-mediated immune tolerance to facilitate infection. Possible strategies of *S. aureus* to affect DCs function are delineated in Figure 3.

## Summary

DCs are vital mediators in both innate and adaptive immune responses. During infection, DCs sense various pathogens through distinct PRRs and orchestrate different immune arms to defend against invading pathogens. However, pathogens such as *S. aureus* have also evolved tactics to evade immune clearance by impairing activation and function of DCs. It is important to further illuminate the role of differing subsets of DCs upon *S. aureus* infection, which may be instrumental in developing new therapeutic strategies for *S. aureus* infection, especially the MRSA infection.

## Abbreviations

DCs: Dendritic cells
APCs: Antigen-presenting cells
PRRs: Pattern recognition receptors
TLRs: Toll-like receptors
PAMPs: Pathogen associated molecular patterns
MHC: Major histocompatibility complex
MRSA: Methicillin-resistant *S. aureus*
CA-MRSA: Community-acquired MRSA
SEs: Staphylococcal enterotoxins
TSST: Toxic shock syndrome toxin-1
ETs: Exfoliative toxins
SR-A: Scavenger receptor A
LCs: Langerhans cells
